# Feasibility of machine integrated point of care lung ultrasound automatic B-lines tool in the Corona-virus 2019 critical care unit

**DOI:** 10.1186/s13054-021-03770-8

**Published:** 2021-09-24

**Authors:** Gal Tsaban, Ori Galante, Yaniv Almog, Yuval Ullman, Lior Fuchs

**Affiliations:** 1 Medical Intensive Care Unit, Soroka University Medical Center, P.O.B 151, Beersheva, Israel; 2 Department of Cardiology, Soroka University Medical Center, Beersheva, Israel; 3 Faculty of Health Sciences, Ben Gurion University of the Negev, Beersheva, Israel

In the last decade, the use of point-of-care lung ultrasound (POC-LUS) has substantially increased and in some intensive care units has largely replaced the routine use of chest X-rays tests [1]. While POC-LUS is highly efficient, it requires expertise, and when wrongfully interpreted may badly influence the treating physician's decision-making. Due to the logistic and medical equipment limitations in quarantined zones in which COVID-19 patients are treated, the importance of POC-LUS increased [2]. We aimed to assess the reliability of a newly designed POC-LUS machine-integrated tool for automatic-real-time bedside quantification of B-lines among critical COVID-19 patients.

We assessed all patients admitted to the COVID-19 intensive-care unit in Soroka University Medical Center (SUMC) in Beersheva between February 2, 2020, and June 23, 2020. POC-LUS was performed daily during the routine medical rounds using Venue_TM_ ultrasound machine, General Electric.

Intensive care specialists acquired data in the COVID-19 ICU by placing the probe longitudinally in the intercostal space and prospectively recording for four seconds. The 4-second time-frame allowed the shortest acquisition time with high sensitivity to detect B-lines [3]. All clips were reviewed and blindly assessed for B-lines quantification by two experienced physician operators (GT, 5 years and LF, 9 years). Separately, post hoc automatic analyses were performed using the Venue_TM_ integrated auto-B-lines tool.

Venue_TM_-auto-B-lines tool provides a grade between zero to ≥ five B-lines, detected only as lines reaching the bottom of the screen. Since the estimation of ≥ 3 B-lines in LUS screening of the lungs' independent zones is considered pathological [4], we also divided the B-lines score to "dry" (≤ 3 B-lines) or "wet" (≥ 4 B-lines).

Each clip was considered an independent observation point. We divided the B-lines assessment into three severity groups: (1) ≤ 2 (non-pathologic), (2) 3–4, and (3) ≥ 5 (severe-ARDS). To assess the agreement between the automatic and physician-assessed quantifications, we performed Cohen's-Kappa tests. A two-sided *p*-value ≤ 0.05 was considered statistically significant. All analyses were performed using SPSS 26.0 (Armonk, NY, USA).

During the study, 153 clips were acquired from ten patients. All clips were interpretable and included in the analysis. Patients were primarily males (90%), with a mean age of 61.0 ± 8.0 and a mean body mass index of 24.5 ± 10.5. The mean-p/f-ratio was 203.7 ± 83.2, and 70% of patients were mechanically ventilated.

When assessed by physician, 104 observations were classified as group 3, 33 as group 2, and 16 as group 1. In automatic analysis, 18 observations were classified as group 1, 33 as group 2, and 102 as group 3. An example of automatic-POC-LUS assessment across the spectrum of SARS-CoV2-ARDS severity is illustrated in Fig. 1. The weighted Cohen's-Kappa for agreement between the automatic and physician quantifications was 0.734 (95% CI 0.641, 0.826; *p* < 0.001). When divided into "dry" (≤ 3 B-lines) vs. "wet" (≥ 4 B-lines), the weighted Cohen's-Kappa was 0.822 (95% CI 0.716, 0.928; *p* < 0.001).

**Fig. 1:**
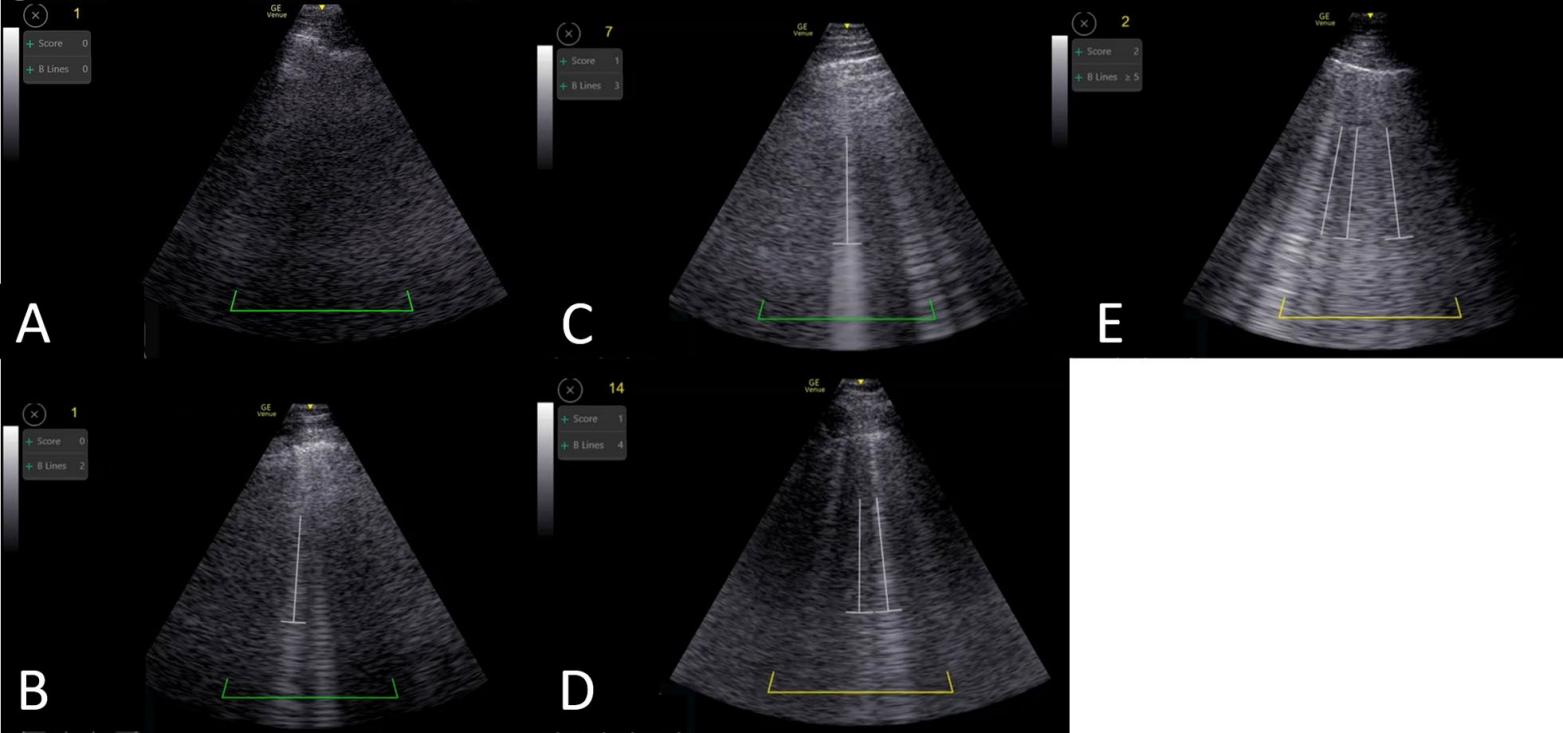
Automatic POC-LUS assessment across the spectrum of ARDS severity. “Dry-lung”: **A** 0 B-lines; **B** 2 B-lines; **C** 3 B-lines. "Wet-lung": **D** 4 B-lines; **E** 5 B-lines

In conclusion, we found that the machine-integrated Venue_TM_-auto-B-lines tool is highly reliable among severe COVID-19 ICU patients. To our knowledge, this is the first study to validate a machine-integrated automatic-B-lines quantification tool with high reliability among COVID-19 patients. The small number of patients included in this feasibility trial should be acknowledged as a limitation; thus, further research to validate the results of this study is warranted. Previously, a non-integrated automatic B-lines detection application has shown good overall reliability while also stressing the difference in assessment reliability dependent on the operator experience [5]. The current study's results may help better interpret POC-LUS assessments performed by less-experienced operators and reduce inter-operator variability. This tool may provide technological infrastructure for future telemedicine, even in non-experienced hands or for self-assessment by patients. The Venue_TM_-auto-B-lines tool may reduce the medical staff's exposure time and promote more accurate and standardized LUS assessment methods.

## Data Availability

All supporting data are available upon request and pending the corresponding author's (LF) approval.
